# Comparative genomic analysis reveals cellulase plays an important role in the pathogenicity of *Setosphaeria turcica* f. sp. *zeae*

**DOI:** 10.3389/fmicb.2022.925355

**Published:** 2022-07-22

**Authors:** Zhoujie Ma, Yufei Huang, Zhaoran Zhang, Xiaodi Liu, Yuanhu Xuan, Bo Liu, Zenggui Gao

**Affiliations:** ^1^Institute of Plant Immunology, College of Plant Protection, Shenyang Agricultural University, Shenyang, China; ^2^College of Life Sciences, Yan’an University, Yan’an, China

**Keywords:** *Setosphaeria turcica*, formae speciales, pathogenicity, genome sequencing, gene function, comparative genome, cellulase activity

## Abstract

*Setosphaeria turcica* f. sp. *zeae* and *S. turcica* f. sp. *sorghi*, the two formae speciales of *S. turcica*, cause northern leaf blight disease of corn and sorghum, respectively, and often cause serious economic losses. They have obvious physiological differentiation and show complete host specificity. Host specificity is often closely related to pathogen virulence factors, including secreted protein effectors and secondary metabolites. Genomic sequencing can provide more information for understanding the virulence mechanisms of pathogens. However, the complete genomic sequence of *S. turcica* f. sp. *sorghi* has not yet been reported, and no comparative genomic information is available for the two formae speciales. In this study, *S. turcica* f. sp. *zeae* was predicted to have fewer secreted proteins, pathogen-host interaction (PHI) genes and carbohydrate-active enzymes (CAZys) than *S. turcica* f. sp. *sorghi*. Fifteen and 20 polyketide synthase (PKS) genes were identified in *S. turcica* f. sp. *zeae* and *S. turcica* f. sp. *sorghi*, respectively, which maintained high homology. There were eight functionally annotated effector protein-encoding genes specifically in *S. turcica* f. sp. *zeae*, among which the encoding gene *StCEL2* of endo-1, 4-β-D-glucanase, an important component of cellulase, was significantly up-regulated during the interaction process. Finally, gluconolactone inhibited cellulase activity and decreased infection rate and pathogenicity, which indicates that cellulase is essential for maintaining virulence. These findings demonstrate that cellulase plays an important role in the pathogenicity of *S. turcica* f. sp. *zeae*. Our results also provide a theoretical basis for future research on the molecular mechanisms underlying the pathogenicity of the two formae speciales and for identifying any associated genes.

## Introduction

Northern leaf blight caused by *Setosphaeria turcica* is a major disease of gramineous crops and leads to serious yield losses of cereal crops in the world, especially during the growing season when the temperature is moderate (15°C–25°C) and the dew is heavy (Ramathani et al., 2011; Galiano-carneiro and Miedaner, 2017). Under natural conditions, *S. turcica* infects a broad range of plants, including corn, sorghum, Sudan grasses, and other sorghum species (Robert, 1960; Bhowmik and Prasada, 1970; Martin et al., 2011). Mitra (1923) first reported a clear physiological differentiation of *S. turcica*, with different formae speciales. *S. turcica* is classified as *S. turcica* f. sp. *zeae*, *S. turcica* f. sp. *Sorghi*, and *S. turcica* f. sp. *complexa*, based on the pathogen infecting a specific host or group of hosts and producing the typical spots. *S. turcica* f. sp. *zeae* can only infect corn, *S. turcica* f. sp. *sorghi* is virulent to sorghum and Sudan grasses, whereas *S. turcica* f. sp. *complexa* infects more plants (Bergquist and Masias, 1974).

Previous studies on *S. turcica* mainly focused on strains isolated from corn (*S. turcica* f. sp. *zeae*). With the completion of the genome sequencing of *S. turcica* f. sp. *zeae* in recent years (Ohm et al., 2012), considerable genetic information became available to understand its infection mechanism and its interaction with corn. At present, molecular-level studies on the pathogenicity of *S. turcica* f. sp. *zeae* mainly focus on signal transduction pathways and extracellular secretions of pathogens, such as cell wall degrading enzymes, host-specific toxin, and melanin (Cuq et al., 1993; Degefu et al., 2004; Ni, 2004). Previous studies have shown that HT (from *Helminthosporium turcicum*) toxin can induce typical symptoms of northern leaf blight in corn. Further, 1, 8-dihydroxynaphthalene (DHN) melanin has been shown to be closely related to pathogenicity (Butler et al., 2001; Nosanchuk and Casadevall, 2003). The secretion of melanin promotes the production of adhesive cells and increases the turgor pressure, which enhances the penetration of *S. turcica* into the corn tissue (Lagunas-Muñoz et al., 2006). Many genes (*StLAC2*, *StPKS* and *St4HNR*) have been proved to play important roles in the melanin synthesis pathway (Wen et al., 2008; Zhang et al., 2011; Ma et al., 2017). Some oxidoreductases are involved in various physiological metabolic activities of pathogens. Deletion of peroxisomes might interfere with the development of pathogenic fungi, reduce virulence, and decrease the ability to resist plant defense enzymes (Segmüller et al., 2008). However, the virulence factors of *S. turcica* f. sp. *sorghi* have not yet been investigated at the molecular level.

The two formae speciales of *S. turcica* could not be distinguished by morphology and internal transcribed spacer (ITS), but inoculation results showed that *S. turcica* f. sp. *zeae* and *S. turcica* f. sp. *sorghi* have high specificity on host and have no obvious cross-infection (Tang et al., 2014). Meanwhile, microsatellites had also been used to distinguish *S. turcica* from corn and sorghum (Nieuwoudt et al., 2018). With the advent of new molecular research techniques, the study of pathogens has remarkably benefited from the information of the genome and the analysis of comparative genomics. A comparative genomic study was conducted on the two pathogens in corn, *Ustilago mayais* and *Sporisorium reilianum*; 43 variant regions were identified in the two species. These regions mainly encode secretory effectors and some virulence clusters (Schirawski et al., 2010). The specialized secondary metabolites and small secretory protein effectors of pathogens are closely related to host specificity (Buiate et al., 2017). *Alternaria longipes* and *A. alternata* can also cause tobacco brown spot disease, but comparative genomic analysis revealed that *A. longipes* has more plant-pathogen-associated genes, carbohydrate-active enzymes (CAZys), secreted protein genes and conditionally dispensable chromosomes (Hou et al., 2016). Therefore, exploring the differences in pathogenicity mechanism between the formae speciales of *S. turcica* and host interaction requires the genomic information of the two formae speciales.

It was reported that pathogens can secrete a large amount of cellulase during the pathogenesis process, which softens the host cell wall leading to faster infection rates and longer disease duration (Wanjiru et al., 2002). In cellulase-inhibited mutants of *Erwinia carotovora* subsp. *carotovora*, this process of cell wall softening was significantly reduced in potato tissues (Walker et al., 1994). Owing to the secretion of other cell wall degrading enzymes, the pathogenicity of *Cochliobolus carbonum* was not affected by the destruction of the *CEL1* gene (Sposato et al., 1995). Furthermore, a highly aggressive strain of *Phaeosphaeria nodorum* secreted more cellulase than a weakly aggressive strain (Lalaoui et al., 2008). The cellulase activity of *S. turcica* f. sp. *zeae* was slightly higher than that of *S. turcica* f. sp. *sorghi*, and the cellulase genes of *S. turcica* f. sp. *zeae* and *S. turcica* f. sp. *sorghi* were significantly upregulated at 72 and 36 h after inoculation, respectively (Tang et al., 2015). D-glucono-l, 5-lactone, a mixed inhibitor of cellulase activity in *Trichoderma reesei*, mainly affects the activity of glucosidase, but also has a inhibitory effect on the activity of exoglucanase, endoglucanase, and related enzymes, and it can induce cellulase gene expression (Reese et al., 1971; Kou et al., 2014).

Obvious host specializations are noted in *S. turcica* f. sp. *zeae* and *S. turcica* f. sp. *sorghi*. The whole genome sequencing and comparative genomic analysis of the two formae speciales of *S. turcica* can help in the identification of the relevant pathogenic genes that help in host-specific interactions; the whole genome sequence of *S. turcica* f. sp. *zeae* has been published in 2012 (Ohm et al., 2012). In the present study, a strain named GD003 was isolated from sorghum leaves infected with northern leaf blight by using the monospore separation method, and then identified using Koch’s postulates and ITS sequencing of *S. turcica* f. sp. *sorghi*. Whole-genome sequencing and gene function annotations revealed important genomic information about *S. turcica*. Comparative genomic analysis revealed differences in the genomes between the two formae speciales, including secreted proteins, pathogen-host interaction (PHI) genes, CAZys and secondary metabolic pathways. Furthermore, we used gluconolactone to alter the pathogenicity of *S. turcica* f. sp. *zeae* and speculated that the cellulase was one of the important reasons for its pathogenicity. The study findings might provide important theoretical information for the pathogenic differentiation mechanism of the formae speciales of *S. turcica* and provide an effective reference for the prevention of northern leaf blight and genetic breeding of resistant varieties.

## Materials and methods

### Fungal isolation and identification

Strain GD003 was isolated from sorghum leaves infected with northern leaf blight by using the single-spore isolation method (Gao et al., 2010). The spores were transferred to water agar (WA: 17 g agar and 1 L ddH_2_O) by tapping the leaves, and then the single spore was directly picked up under low magnification and transferred to potato dextrose agar (PDA: 200 g potato, 20 g glucose, and 17 g agar, and 1 L ddH_2_O) by using a simple homemade needle. The strain GD003 was deposited in the Institute of Plant Immunology, Shenyang Agricultural University and used to study pathogenic mechanism of the pathogen for 5 years. The strain was incubated under continuous darkness at 25°C.

ITS sequences and the inoculation and were used for the identification of strain GD003. Mycelia were collected from potato dextrose broth, the DNA was separated using the modified CTAB method, and the ITS sequences were amplified using PCR by using primers ITS1 and ITS4 (Gardes and Bruns, 1993; Okori et al., 2004). The amplified product was sequenced, and phylogenetic relationships were analyzed using MEGA4.0 (Tamura et al., 2007) as well as the neighbor-joining (NJ) model. Bootstrap replication was set to 1,000, and the bootstrap value was at the branch node. Sweet sorghum variety LR115 and corn variety Huobai susceptible to northern leaf blight were obtained from Dr. Jiang (Liaoning Academy of Agricultural Sciences, China). Three germinated seeds were sown in pots having 15 cm diameter and cultivated in a greenhouse with a temperature of 21/18°C day/night and light intensities of 35–50 Klux. When the plants grew to the V6 stage, the strain incubated for 2 weeks was added to a small amount of sterile water and filtered through a double-layered gauze to form a suspension of 1 × 10^6^ conidia per milliliters. Tween-20 was added to the prepared spore suspension to a final concentration of 0.1%, and the seedlings were inoculated the spore suspension by using a sprayer; after inoculation, the seedlings were transferred to a plastic shed for 48 h for moistening, and then transferred to a greenhouse. The leaves of plants were inspected for symptoms of infection at 14 days after inoculation.

### Genome sequencing and assembly

The improved CTAB method was used to extract genomic DNA from GD003, a sorghum-specific strain of *S. turcica*. After DNA was qualified by electrophoresis, two DNA libraries were constructed, of which 350 bp small fragment library was sequenced at paired-end by HiSeq PE150 and 20 kb SMRT Bell library was sequenced at single-molecule by PacBio RSII. Sequencing was performed at the Beijing Novogene Bioinformatics Technology Co., Ltd. (Beijing, China). The low quality reads were filtered by the SMRT Link v5.0.1 (Li et al., 2010) and the filtered reads were assemblied to generate contigs. The relationship between the contigs were determined by SOAPdenovo2 (Luo et al., 2012) to obtain the final assembly results that reflecting the basic conditions of the sample genome, including total data, GC content, read coverage depth, and mass value distribution.

### Comparative genomic analysis

The genome sequences of *S. turcica* f. sp. *zeae* Et28A was deposited at joint genome institute (JGI) with project ID 401988. The open reading frames across the genome were predicted and filtered using Augustus software (Stanke et al., 2006), and the number, total length, average length, and proportion of encoding genes were recorded. The gene function annotation was mainly based on the comparison of protein sequences, and the local comparison tool BlastP (Gao et al., 2011) was used for homology matching with the annotation results on GenBank, gene ontology (GO; Ashburner et al., 2000), kyoto encyclopedia of genes and genomes (KEGG; Kanehisa et al., 2004), cluster of orthologous groups (COG; Tatusov et al., 2003), non-redundant protein sequence (NR; Li et al., 2002), transporter classification database (TCDB; Milton et al., 2009), Swiss-Prot (Bairoch and Apweiler, 2000), PHI (Urban et al., 2015) and CAZy (Cantarel et al., 2009) databases to obtain the corresponding functional annotation information. SignalP (Petersen et al., 2011) was used to analyze the N-terminal signal peptide of encoded proteins, TMHMM (Krogh et al., 2001) was used for transmembrane structure prediction, and TargetP (Emanuelsson et al., 2007) was used to predict the subcellular localization of encoded proteins. Proteins located extracellularly, with signal peptide and lacking transmembrane domains, were defined as secreted proteins. Further, effectors in secreted proteins were screened by EffectorP (Sperschneider et al., 2016).

The key genes for secondary metabolite syntheses were identified using antiSMASH v4.0.2 program (Medema et al., 2011), especially polyketide synthase (PKS) coding genes. The MEGA4.0 (Tamura et al., 2007) was used to compare the protein domains encoded by PKSs of two formae speciales and other plant pathogenic fungi, and to construct phylogenetic tree, including *Bipolaris maydis* T-toxin synthesis related to PKS1 (GenBank accession number: AAB08104), *Fusarium graminearum* zearalenone synthesis related to PKS4 (GenBank accession number: ABB90283), *Aspergillus nidulans* locastatin synthesis related to LovF (GenBank accession number: AAD34559), *F. verticillioides* fumonisin synthesis related to Fum1p (GenBank accession number: AAD43562), *A. steynii* ochratoxin synthesis related to PKS (GenBank accession number: AHZ61902), and *A. alternate* ACT-toxin synthesis related to ACTTS3 (GenBank accession number: BAJ14522), as well as genes closely related to melanin synthesis such as *B. oryzae* PKS1 (GenBank accession number: BAD22832), *S. turcicia* PKS (GenBank accession number: AEE68981), *Ascochyta rabiei* PKS1 (GenBank accession number: ACS74449), *Podospora anserine* PKS1 (GenBank accession number: CDP25014), *Colletotrichum lagenarium* PKS1 (GenBank accession number: BAA18956), *A. fumigatus* A1b1p (GenBank accession number: ACJ13039), *Ceratocystis resinifera* PKS1 (GenBank accession number: AAO60166).

### Real-time PCR analysis of genes encoding the specific effectors of *Setosphaeria turcica* f. sp. *zeae*

The mycelium disk of strain Et28A with diameter of 1 cm was inoculated onto corn leaves *in vitro*, and 50–100 mg of the leaves under the disk were cut with RNA-free scissors at different infection periods (0, 24, 48, 72, and 96 h), wrapped in tin foil and immediately frozen in liquid nitrogen for 10 min, transferred to −80°C for storage. Total cellular RNA was isolated using an Ultrapure RNA Kit (CWBIO, Beijing, China), and then cDNA was synthesized using the PrimeScript™ RT reagent Kit with gDNA Eraser (Perfect Real Time; TaKaRa, Tokyo, Japan). The reaction mixture contained 10 μl of TB Green Premix Ex Taq II (Tli RhaseH Plus; TaKaRa, Tokyo, Japan), 1 μl of forward primer, 1 μl of reverse primer, 2 μl of cDNA, and 6 μl of ddH_2_O. The reactions were performed in the CFX-96 system (BioRad, Hercules, CA, United States), and all samples were tested in triplicate and repeated twice. All reaction conditions were performed as follows: initial denaturation at 95°C for 30 s, followed by 40 cycles of denaturation at 95°C for 5 s and annealing at 60°C for 30 s. The cycle threshold (Ct) values were analyzed using CFX Manager and relative expression levels of functionally annotated effector protein-coding genes specific for *S. turcica* f. sp. *zeae* were calculated at each period according to the 2^-△△Ct^ method.

### Effect of gluconolactone on *Setosphaeria turcica* f. sp. *zeae*

The gluconolactone solution was sterilized and cooled, and then added to a PDA medium under sterile conditions to final concentrations of 0.2, 0.4, and 0.8% (w/v). Five millimeter agar disks containing mycelium of strain Et28A were transferred to the medium with an inoculation needle. Then the side of the hyphae was pressed down to the medium and one agar disk was placed in the center of each dish. Each treatment was repeated five times and cultured at 25°C for 5 days. The gluconolactone solution was replaced by sterile water for the control group. For each treatment group, the colony diameter was measured then the growth inhibition rate was calculated (Quiroga et al., 2001).

To analyze the effect of gluconolactone on cellulase activity, preparation of the crude enzyme solution was slightly modified based on the methods of Lee and Blackburn (1975). The agar disks containing mycelium of strain Et28A that were cultured on the PDA were added to Czaper liquid culture medium (2 g KNO_3_, 0.5 g KCl, 0.01 g FeSO_4_, 1 g K_2_HPO_4_, 0.5 g MgSO_4_, 10 g sodium carboxymethyl cellulose, and 1 L ddH_2_O) both with and without gluconolactone (0.2, 0.4, and 0.8%; w/v). Then, nine disks were inoculated in 150 ml medium and were shaken for 1 h per day (120 rpm), incubated for 15 days at 25°C in the dark, and then filtered through sterile double gauze (22 mesh). The filtrate was centrifuged at 4°C and 10,000 g for 20 min and the crude enzyme solution was the supernatant. Cellulase activity was determined based on the method described by Eveleigh et al. (2009). Briefly, 1 ml of 1% sodium carboxymethyl cellulose in 0.1 M citrate buffer (pH 4.5) and 0.5 ml extracted crude enzyme solution were placed in a test tube and then incubated in a 50°C water bath for 30 min. After the reaction mixture was cooled, 3 ml of 3, 5-dinitrosalicylic acid reagent was added, and the solution was heated to 100°C for 5 min. The absorbance at 540 nm of the reaction mixture after appropriate dilution was measured with a spectrophotometer. A cellulase activity unit (U) was defined as the amount of enzyme required to catalyze the reaction to produce 1 μmol of reducing sugar per min under specific conditions. All enzyme activity assays were repeated three times. The protein content was determined using the Coomassie brilliant blue G250 staining method (Bradford, 1976).

Analysis of expression levels was used to measure the effect of gluconolactone on endo-1, 4-β-D-glucanohydrolase encoding gene A2464. The agar disks containing the mycelium of *S. turcica* f. sp. *zeae* Et28A that were cultured on the PDA were inoculated *in vitro* on corn leaves at the 6–8 leaf stage. One hundred microliter gluconolactone solutions (0.2 and 0.4%, w/v) were added to the edge of the disks each day, and the control group was treated with sterile water. The expression levels of A2464 were determined at different infection periods (0, 6, 12, 24, 36, 48, 72, and 96 h). All samples were tested in triplicate and repeated twice. Finally, the infection rate and pathogenicity were determined and the inoculation method was described above. Pathogenicity was determined after culturing for 72 h in the dark at 25°C for moistening. Then it was stained with trypan blue, dehydrated with saturated chloral hydrate, and rinsed with sterile water before being placed under a microscope to observe the infection efficiency of *S. turcica* f. sp. *zeae* Et28A. Each treatment was repeated three times.

### Statistical analysis

All statistical tests were calculated in SPSS Statistics 19 software. Data were represented as means ± standard error of at least three repeated experiments. *p* < 0.05 was defined as a statistically significant difference.

## Results

### Identification of *Setosphaeria turcica* f. sp. *sorghi* GD003

**Figure 1:**
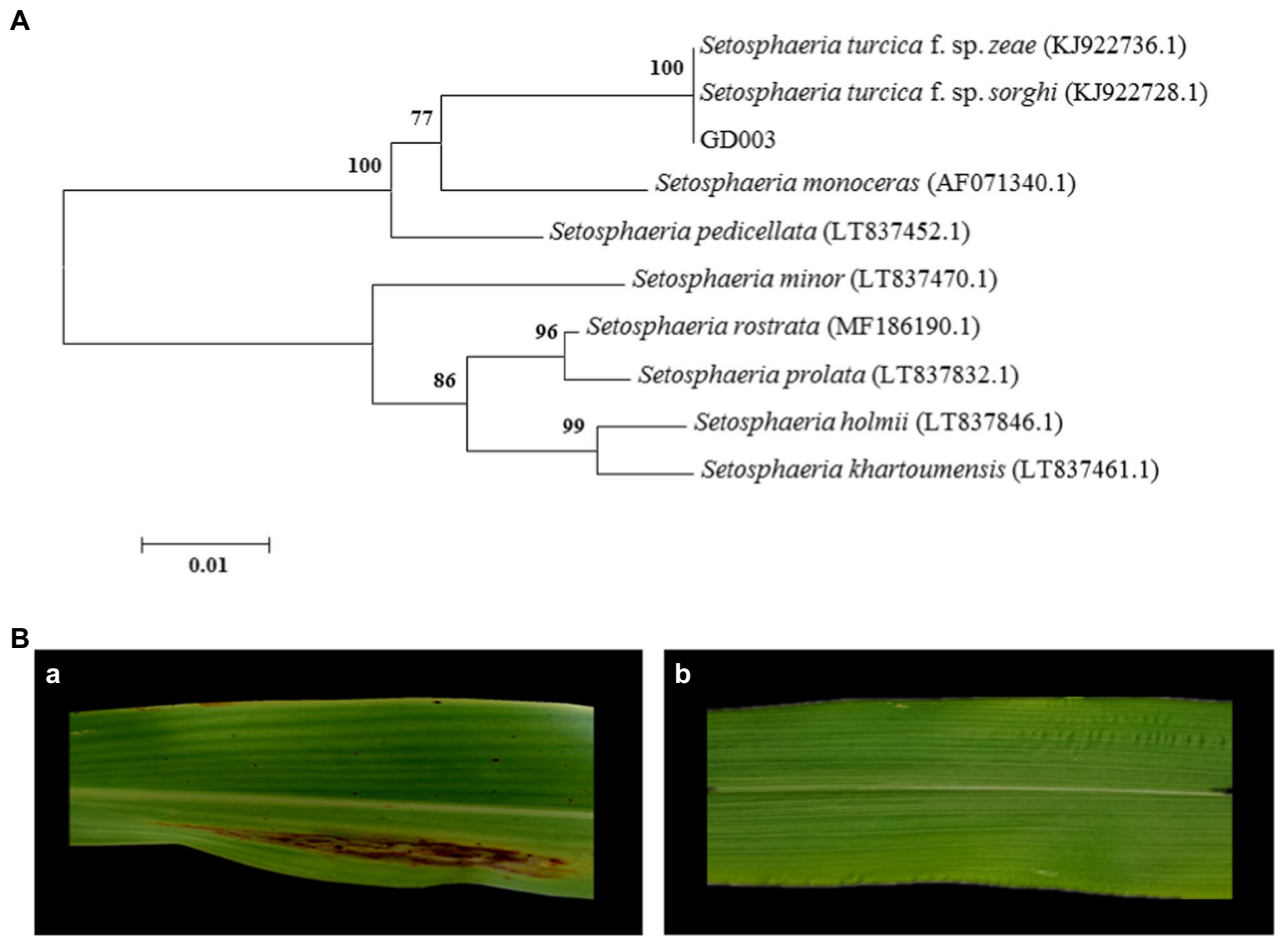
Identification of GD003 based on ITS sequences and pathogenicity. **(A)** Phylogenetic analysis of ITS sequences of GD003. The numbers in parentheses indicate the accession numbers of ITS sequences of the species in GenBank. **(B)** Pathogenicity of GD003 strain on sorghum and corn. **(a)** Typical lesions of GD003 on sorghum. **(b)** No visible reaction of GD003 on corn.

Phylogenetic tree of the ITS sequences showed that GD003, *S. turcica* f. sp. *zeae* strain QDY1307 (GenBank accession number: KJ922736.1) and *S. turcica* f. sp. *sorghi* strain LLG1302 (GenBank accession number: KJ922728.1) were in the same branch (only four differential bases; Figure 1A). The ITS sequences do not distinguish the two formae speciales of *S. turcica.* Within 14 days after inoculation, this GD003 strain formed a typical long spindle lesion on sorghum leaves, while no visible reaction was evident on corn leaves (Figure 1B), so the pathogenicity tests identified strain GD003 as *S. turcica* f. sp. *sorghi*.

### Genomic sequencing and assembly of *Setosphaeria turcica* f. sp. *sorghi* GD003

After electrophoresis, 159.60 ng/ml DNA yielded OD260/280 of 1.87 and OD260/230 of 2.24; the fragment size was mainly distributed above 30 K, and the genome was slightly broken, which met the requirements for single-sequencing database creation. A total of 7.82 Gb of reads were obtained by sequencing the genome of *S. turcica* f. sp. *sorghi* GD003 (depth: 177×), including 938,546 reads. The length of the sequence N50 was 11,965 bp, and the average sequencing quality value was 0.86. The genome assembly revealed 22 contigs (Supplementary Fasta 1) with a total length of 44,063,561 bp and a GC content of 50.7%. The scatter diagram of *S. turcica* f. sp. *sorghi* GD003 genomic GC-depth was mostly concentrated in the range of 40%–60% (Supplementary Figure 1).

### Genome comparison of two formae speciales of *Setosphaeria turcica*

A total of 10,428 protein-coding genes (Supplementary Fasta 2) were predicted in the genome of *S. turcica* f. sp. *sorghi* GD003, accounting for 35.47% of the total length of the genome sequence, and the average length of the coding genes was 1,499 bp. In contrast, only 8,276 protein-coding genes (Supplementary Fasta 3) were found in the genome of *S. turcica* f. sp. *zeae* Et28A, accounting for 26.86% of the total length of the genomic sequence, and the average length of the coding genes was 1,396 bp (Table 1). From the gene distribution map, the most abundant *S. turcica* f. sp. *sorghi* GD003 and *S. turcica* f. sp. *zeae* Et28A genes were found to be concentrated in the region of 2,500 bp or more, including 1,431 and 882 genes, respectively.

**Table 1:** *Setosphaeria turcica* f. sp. *sorghi* GD003 and *S. turcica* f. sp. *zeae* Et28A genome features.

Features	*S. turcica* f. sp. *sorghi* GD003	*S. turcica* f. sp. *zeae* Et28A
Genome size (bp)	44,063,561	43,014,577
Gene number	10,428	8,276
Gene length (bp)	15,630,591	11,553,107
Gene GC content (%)	55.94	56.52
% of genome (genes)	35.47	26.86
Gene average length (bp)	1,499	1,396
Gene internal length (bp)	28,432,970	31,461,470
Gene internal GC content (%)	47.83	49.57
% of genome (internal)	64.53	73.14

Different numbers of genes in the two formae speciales genomes were annotated in each functional database (Table 2). By comparing *S. turcica* f. sp. *sorghi* GD003 and *S. turcica* f. sp. *zeae* Et28A, 704 and 521 secreted proteins were predicted, respectively (Supplementary Figure 2), containing 161 and 137 effectors, which were required for the pathogens to act directly or indirectly on the hosts. The findings suggested that the *S. turcica* f. sp. *sorghi* GD003 and *S. turcica* f. sp. *zeae* Et28A could directly secrete 42 and 33 small cysteine-rich proteins (SCRPs; the number of amino acids is less than or equal to 200, and cysteine content is 4% or more), respectively, of which 30 SCRPs existed in both formae speciales.

**Table 2:** Number of genes annotated by different functional databases in *Setosphaeria turcica* f. sp. *sorghi* GD003 and *S. turcica* f. sp. *zeae* Et28A genomes.

Database type	*S. turcica* f. sp. *sorghi* GD003		*S. turcica* f. sp. *zeae* Et28A	
	Number	Percent (%)	Number	Percent (%)
GO	7,000	67.13	5,708	68.97
KEGG	9,570	91.77	7,762	93.79
COG	2048	19.64	2043	24.69
NR	10,161	97.44	8,013	96.82
TCDB	428	4.10	411	4.97
SwissProf	3,039	29.14	2,952	35.67
PHI	796	7.63	673	8.13
CAZy	480	4.60	442	5.34
Secretory protein	704	6.75	521	6.30

In the present study, 796 and 673 PHI genes were detected in *S. turcica* f. sp. *sorghi* GD003 and *S. turcica* f. sp. *zeae* Et28A, covering 609 and 539 PHI accessions, respectively, but they had seven types of phenotypic mutations (Supplementary Table 1). The PHI information of 655 genes was identical in the two formae speciales. Among 141 PHI genes unique to *S. turcica* f. sp. *sorghi* GD003, excluding 81 genes that did not affect the pathogenicity, phenotypic mutants of 45 genes (accession number: PHI139, PHI323, PHI339, PHI3837, PHI3865, PHI4992, etc.) had reduced virulence. Further searches revealed that the secreted proteins of *S. turcica* f. sp. *sorghi* GD003 and *S. turcica* f. sp. *zeae* Et28A contained 62 and 54 PHI related genes, respectively, of which 51 PHI genes were homologous and 11 PHI genes were specific in *S. turcica* f. sp. *sorghi* GD003.

**Figure 2:**
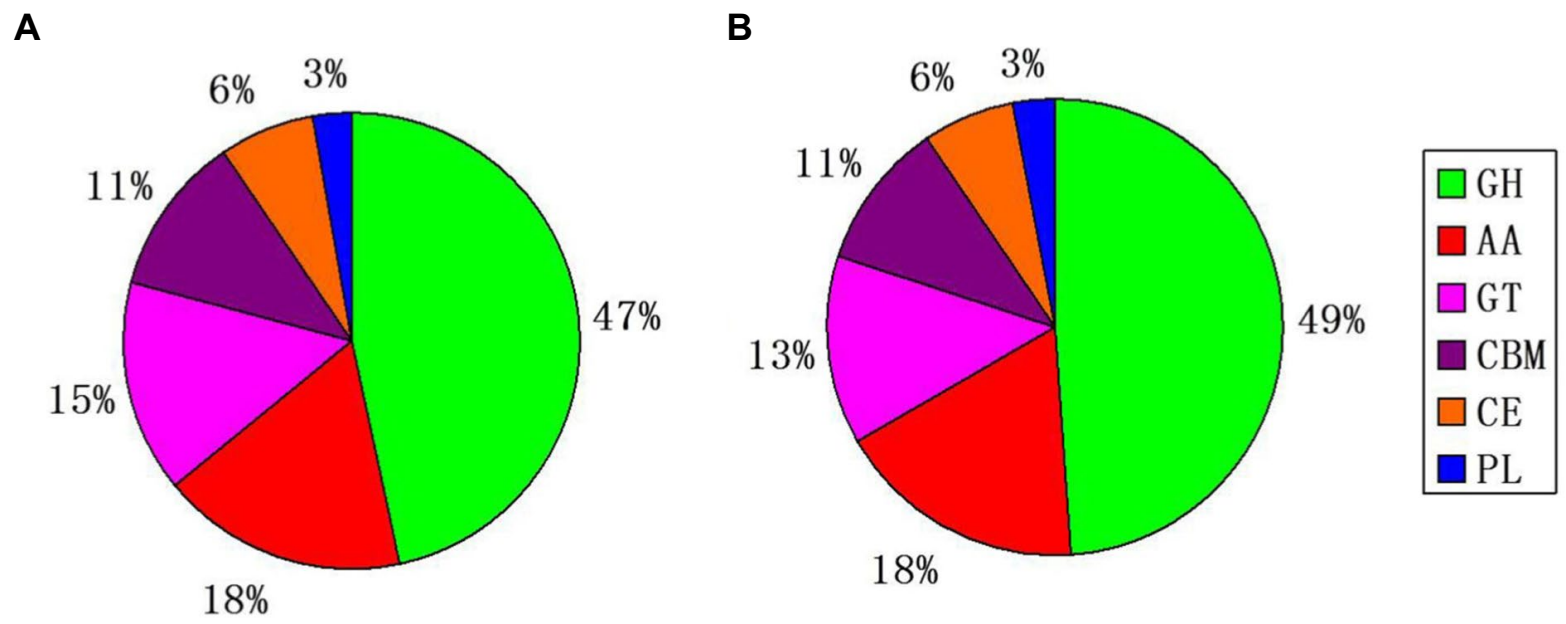
Classification and comparison of the two formae speciales by using whole genome CAZy database. **(A)** *Setosphaeria turcica* f. sp. *sorghi* GD003. **(B)** *S. turcica* f. sp. *zeae* Et28A. GH, glycoside hydrolase; AA, auxiliary activity; GT, glycosyltransferase; CBM, carbohydrate-binding module; CE, carbohydrate esterase, and PL, polysaccharide lyase.

Blastp alignment was performed using the genomically encoded proteins and CAZy database, and 480 and 442 CAZys were identified from *S. turcica* f. sp. *sorghi* GD003 and *S. turcica* f. sp. *zeae* Et28A, respectively; the related CAZys were mainly involved in carbohydrate degradation, modification, and biosynthesis (Figure 2). The most common CAZys were glycoside hydrolases (GHs) containing 224 and 216 genes, respectively, and the remaining were auxiliary activities (AAs), glycosyltransferases (GTs), carbohydrate binding modules (CBMs), carbohydrate esterases (CEs) and polysaccharide lyases (PLs). Further analysis found that 216 (30.68%) and 178 (34.17%) CAZy genes were identified in the secreted proteins of *S. turcica* f. sp. *sorghi* GD003 and *S. turcica* f. sp. *zeae* Et28A, respectively, and most of these genes were associated with GHs in the subfamily classification (Supplementary Table 2).

**Figure 3:**
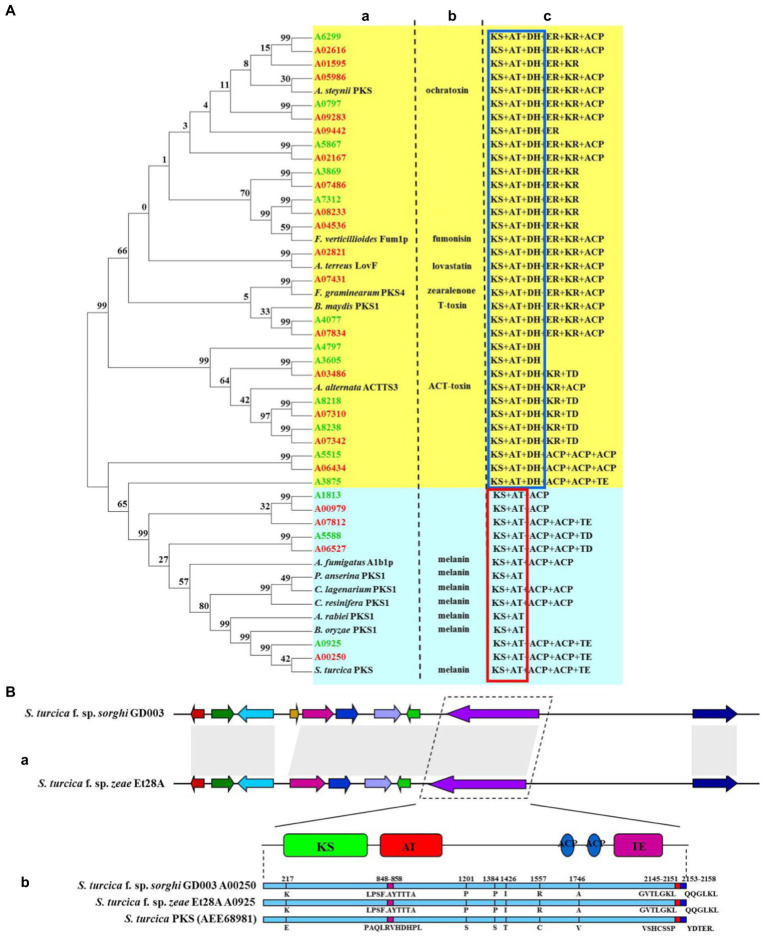
Comparative analysis of polyketide synthase (PKS) key genes of two formae speciales of *Setosphaeria turcica*. **(A)** Phylogeny and domain comparison of PKSs from *Setosphaeria turcica* f. sp. *sorghi* GD003 and *S. turcica* f. sp. *zeae* Et28A with that from other known plant pathogenic fungi. (a) Phylogenetic analysis of proteins encoded by PKS genes. (b) PKS-related toxin and melanin from other known phytopathogenic fungi. (c) PKS-associated protein domains. KS, ketoacyl synthase; AT, acyltransferase; DH, dehydratase; ER, enoylreductase; KR, ketoacyl reductase; ACP, acyl carrier protein; TE, thioesterase; and TD, tudor domain. Words marked in green and red indicate gene names for *S. turcica* f. sp. *zeae* Et28A and *S. turcica* f. sp. *sorghi* GD003, respectively. The red and blue boxes indicate common domains. **(B)** Comparison of gene clusters and core gene-coding sequences of melanin synthesis in *S. turcica* f. sp. *sorghi* GD003 and *S. turcica* f. sp. *zeae* Et28A genomes. (a) Melanin synthesis gene clusters of the two formae speciales. (b) Differences in protein sequence of the melanin synthesis core gene from known *S. turcica*.

Further, 20 and 15 PKSs were predicted for secondary metabolic gene clusters in *S. turcica* f. sp. *sorghi* GD003 and *S. turcica* f. sp. *zeae* Et28A genomes, respectively (Supplementary Table 3). The PKS genes of the two formae speciales were mainly divided into two types (Figure 3A). The core domain of type I consisted of ketoacyl synthase (KS), acyltransferase (AT) and dehydratase (DH), including 16 *S. turcica* f. sp. *sorghi* GD003 PKSs, 12 *S. turcica* f. sp. *zeae* Et28A PKSs, and 6 other PKSs related to the synthesis of phytopathogenic mycotoxins. The other type of core domain was KS + AT, including 4 *S. turcica* f. sp. *sorghi* GD003 PKSs, 3 *S. turcica* f. sp. *zeae* Et28A PKSs, and 7 known melanin synthesis-related PKSs from phytopathogenic fungi. Further analysis of PKSs associated with melanin synthesis revealed that the two formae speciales shared the same core domain, including one KS, one AT, two acyl carrier proteins (ACPs) and one thioesterase (TE), and both coding sequences were 99.48% similar to the known *S. turcica* PKS (GenBank accession number: AEE68981; Figure 3B).

### Analysis of the expression levels of *Setosphaeria turcica* f. sp. *zeae* specific effector coding genes

**Figure 4:**
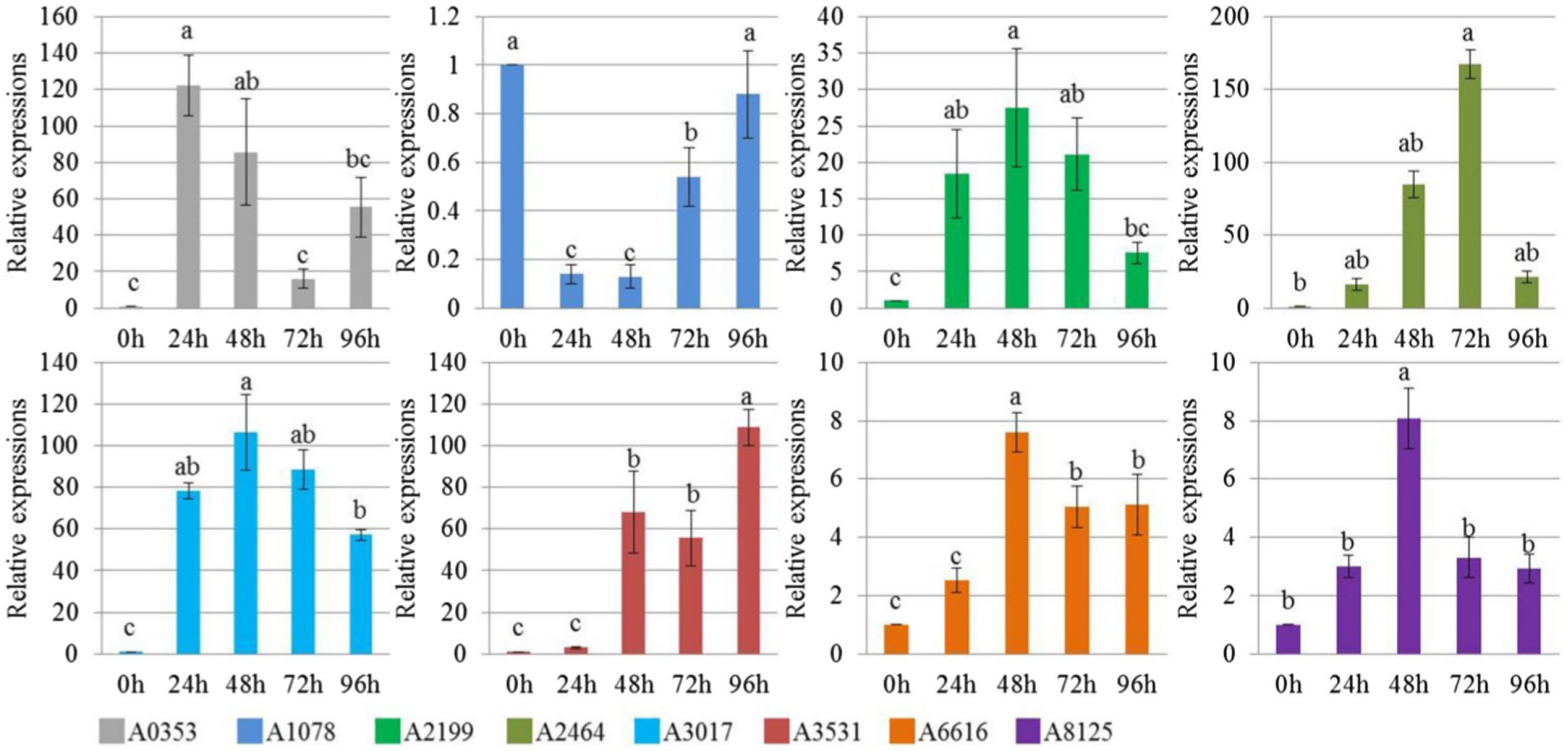
Relative expression level of *Setosphaeria turcica* f. sp. *zeae* specific effector coding genes at different infection periods postinoculation. Error bars represent means ± SE of three repeated experiments (*n* = 3). Different letters indicate significant differences (*p* < 0.05). A0353 encodes carbohydrate binding module family 18 protein; A1078 encodes glycoside hydrolase family 20 protein; A2199 encodes carbohydrate esterase family 1 protein; A2464 encodes glycoside hydrolase family 7 protein; A3017 encodes Argonaute/Dicer protein PAZ; A3531 encodes glycoside hydrolase family 62 protein; A6616 encodes glycoside hydrolase family 16 protein; A8125 encodes glycoside hydrolase family 10 protein.

In our study, 21 effector protein-coding genes were found specifically in *S. turcica* f. sp. *zeae* Et28A, and 13 of them were defined as encoding hypothetical proteins. 18S rRNA was used as a reference gene, primer sequences of eight functionally annotated effector protein-encoding genes specific to *S. turcica* f. sp. *zeae* were designed (Table 3). Compared with the 0 h control, only A1078 was downregulated after inoculation, while the expression levels of A0353, A2199, A2464, A3017, A6166, and A8125 were significantly upregulated. The expression level of A2464 after 72 h of inoculation increased by more than 150-fold compared with that before (Figure 4).

**Table 3:** Primer sequences and amplification lengths of functionally annotated effector protein-coding genes in *Setosphaeria turcica* f. sp. *zeae.*

Gene ID	Accession number	Functional description	Primer	Seq (5’to3’)	Amplification length (bp)
A0353	XP_008021443.1	Carbohydrate-binding module family 18 protein	CBM18-F	CCAAGAACGACATCCAGGACCAG	138
			CBM18-R	GACCGCAGCTCTCGCCATTC	
A1078	XP_008022422.1	Glycoside hydrolase family 20 protein	GH20-F	CGCACTGGCAACGGTCCTTAC	178
			GH20-R	GCGGTTGAAGGCGTTGGAGAC	
A2199	XP_008024103.1	Carbohydrate esterase family 1 protein	CE1-F	CAGGCGACAAGGCAGAAGTGG	153
			CE1-R	CTCACGGTTGCCTGGCTGTATC	
A2464	XP_008024638.1	Glycoside hydrolase family 7 protein	GH7-F	GGTGGTCGCTCCAAGCTCAAC	144
			GH7-R	AATCTGAGTCGCCTGGCTGTTG	
A3017	EKG13298.1	Argonaute/Dicer protein PAZ	PAZ-F	GCAACGCCTACGACTTCTTCCTC	121
			PAZ-R	GTCATGGCCTGCATCTGGTCTG	
A3531	XP_008025955.1	Glycoside hydrolase family 62 protein	GH62-F	TACCTGCGAACCTCCGTCCATC	111
			GH62-R	GGTTGCGGCTGTCCTTCTTGG	
A6616	XP_008029324.1	Glycoside hydrolase family 16 protein	GH16-F	AAGTCACGGCAGGAAGCATCAAC	173
			GH16-R	GGATTGGAGAATGGCAGACGACAC	
A8125	XP_008031550.1	Glycoside hydrolase family 10 protein	GH10-F	GCACTGACAATCCGCAATGACAAC	121
			GH10-R	CTTGACTTGGTTCCGTGGCATCC	

### Inhibition effect of gluconolactone on cellulase activity and pathogenicity of *Setosphaeria turcica* f. sp. *zeae*

**Figure 5:**
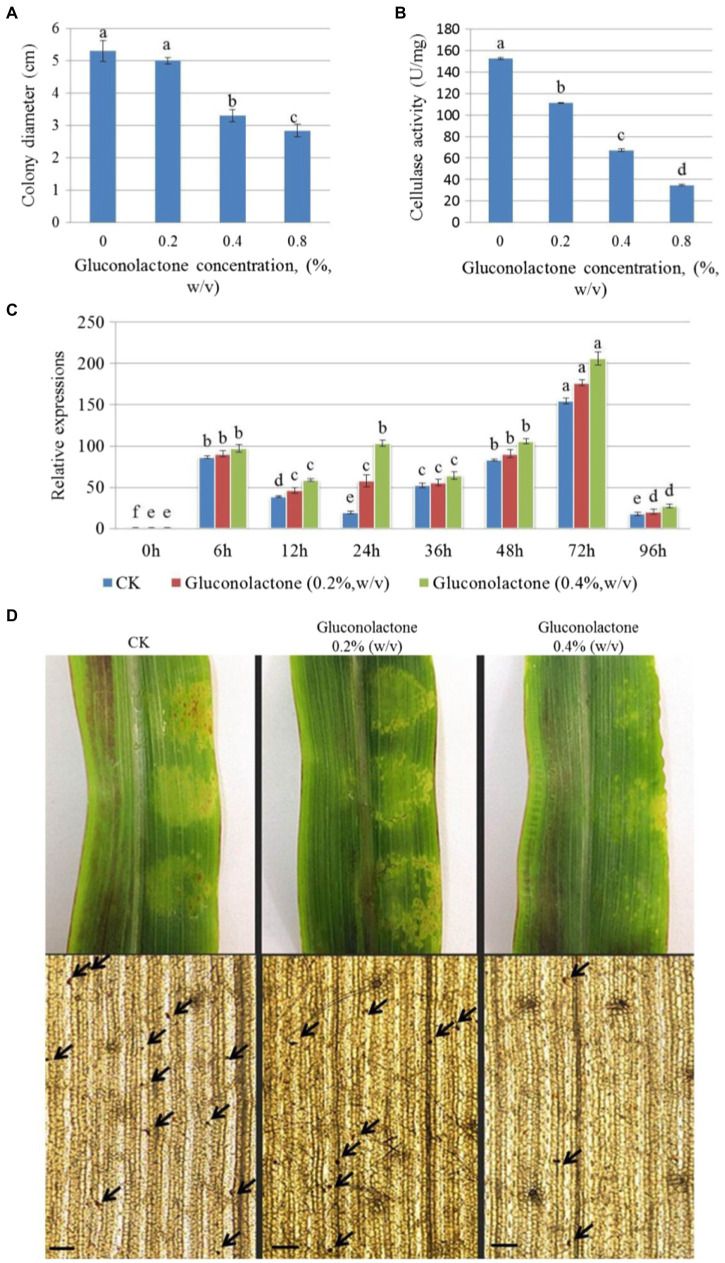
Effect of gluconolactone on *Setosphaeria turcica* f. sp. *zeae*. **(A)** Effect of gluconolactone on colony diameter. Error bars are presented as means ± SE (*n* = 5). Different letters indicate significant differences (*p* < 0.05). **(B)** Effect of gluconolactone on cellulase activity. Error bars indicate means ± SE (*n* = 3). **(C)** Effect of glucolactone on the expression level of endo-1, 4-β-D-glucanase encoding gene *StCEL2*. Data represent means ± SE (*n* = 3). **(D)** Effect of gluconolactone on the pathogenicity and infection rate. Arrow positions are the infection dots. Scale bars are equal to 50 μm.

At a concentration of 0.4% (w/v), hyphal growth was significantly inhibited. When the concentration increased to 0.8% (w/v), the inhibition rate reached 46.12%, which indicated that gluconolactone inhibited the hyphal growth of *S. turcica* f. sp. *zeae* Et28A (Figure 5A). The cellulase activity of *S. turcica* f. sp. *zeae* Et28A was significantly inhibited by different concentrations of gluconolactone (*p* < 0.05), and the effect increased with increasing concentration (Figure 5B). Despite different gluconolactone concentrations (0.2 and 0.4%, w/v), *StCEL2* gene expression showed a consistent trend, all peaked at 72 h. *StCEL2* gene expression level increased with increasing gluconolactone concentration during the same infection period (Figure 5C). Differently-treated pathogens could invade the host and caused corn leaf lesions after 72 h of inoculation. However, the number of invasion sites observed in the control group was significantly higher than that in the gluconolactone treatment group. Furthermore, the number of invasion sites decreased with increasing concentration (Figure 5D), indicating that gluconolactone affected the infection and pathogenicity of *S. turcica* f. sp. *zeae* Et28A.

## Discussion

Previous studies have shown that the variability of ITS sequences among different formae speciales of the same fungus is limited. For example, the ITS and EF-alpha elongation factor analyses cannot identify the formae speciales of *F. oxysporum* (Zhang et al., 2013). Since the ITS information of GD003, *S. turcica* f. sp. *zeae* strain QDY1307 (GenBank accession number: KJ922736.1), and *S. turcica* f. sp. *sorghi* strain LLG1302 (GenBank accession number: KJ922728.1) reveal a difference of only four bases, it proves once again that ITS could not identify the formae speciales of *S. turcica*. Because of the obvious host-specificity between the formae speciales (Mitra, 1923), the inoculation of sorghum leaves showed a clear feature of northern leaf blight. Finally, GD003 was identified as *S. turcica* f. sp. *sorghi* and named *S. turcica* f. sp. *sorghi* GD003.

In this study, we comprehensively reported the first genome information of *S. turcica* f. sp. *sorghi* and compared it with the published genomic data of *S. turcica* f. sp. *zeae* Et28A (JGI ID: 401988), which can provide a reference for revealing the pathogenic mechanism of *S. turcica*. Different strains of the same species also have large differences in genomic structure and encoded proteins (Condon et al., 2013). Several random amplified polymorphic DNA haplotypes uniquely present in sorghum strains of *S. turcica* were not observed in strains collected from corn (Borchardt et al., 1998; Ferguson and Carson, 2004). Genetic differences were confirmed in two formae speciales of *S. turcica* by universally primed polymerase chain reaction (Tang et al., 2014). The genome size of *S. turcica* f. sp. *zeae* GD003 (44.06 Mb) is greater than that of *S. turcica* f. sp. *sorghi* Et28A (43.01 Mb), which may be caused by the pressure of host selection (Thrall et al., 2002). Changes of GC content were speculated to prompt *Curvularia lunata* to mutate more frequently in virulence differentiation (Gao et al., 2014). The GC content of the same species shows a concentrated distribution in the sequencing depth profile (Forsdyke, 1996). Concentration of most of the points in the distribution map in a narrow range (40%–60%) indicates no pollution in the assembly results.

Secreted protein is a generic term for a class of proteins that are produced by cells at specific times and conditions and transported extracellularly, which is often directly related to PHI, is a candidate effector, and is more likely to exhibit population differences during natural selection (Klein et al., 1996). Therefore, studies on secreted proteins might help understand host specificity issues in PHIs. The secreted proteins related to pathogenicity are mainly avirulence genes of pathogens, products of pathogenic genes, and related regulatory proteins (Rep, 2005), such as cell wall degrading enzymes can reduce or even overcome the host’s barrier to pathogen infection (Brito et al., 2006), elicitor substances can induce pathogenic responses in host plants (Kamoun et al., 1998), and some of the secreted proteins can also degrade the antifungal toxin produced by host plants to facilitate the progression of infection (Sperschneider et al., 2016). In this study, more secreted proteins were predicted in *S. turcica* f. sp. *sorghi* GD003. Many phytopathogenic fungi can also directly secrete SCRPs, which have a close relationship with the mechanism of pathogenesis (Rep et al., 2004). These small molecular proteins can act as virulence effector proteins and have carbohydrate-binding activity; they can directly play a role by interfering with host cell signal transduction or inhibiting host PAMP-triggered immunity responses (Marcet-Houben et al., 2012). Twelve SCRPs unique to *S. turcica* f. sp. *sorghi* GD003 were all uncharacterized proteins, and further studies are warranted to determine the function of SCRPs specific to the two formae speciales in PHI.

The genes involved in the interaction between pathogens and hosts play a crucial role in pathogenesis; their products are directly involved in the adaptation and response of pathogens to the host-infecting environment, and the secreted elicitors can directly induce the host plants to express the symptom (Barrett et al., 2009). The PHI database integrates pathogen-related genes to different hosts such as animals, plants, and microorganisms and is widely used to investigate plant pathogen genomes and genes implicated in virulence (Winnenburg et al., 2006; Urban et al., 2015). In this study, 45 genes with phenotypic mutations with reduced pathogenicity were specifically present in *S. turcica* f. sp. *sorghi* GD003, such as PHI139 was required for *Cryptococcus neoformans* to maintain virulence (Chang and Kwon-chung, 1999), the loss of PHI339 significantly reduced the pathogenicity of *C. lindemuthianum* (Siriputthaiwan et al., 2005), and PHI4992 was required for *Candida albicans* biofilm formation *in vitro* and *in vivo* (Desai et al., 2015). Differences in these PHI-related genes might lead to differences in pathogenicity between the two formae speciales. Significantly, there were 11 PHI genes specific for the secreted protein encoding genes of *S. turcica* f. sp. *sorghi* GD003, the knockout of PHI323 significantly reduced the virulence of *Verticillium fungicola* (Amey et al., 2003), and PHI3865 was required for *Penicillium expansum* to cause blue mold rot (Barad et al., 2012), while the related genes PHI569, PHI2849, and PHI6126 reported in *Fusarium* did not affect their pathogenicity.

The plant pathogenic fungi CAZys play a crucial role in degrading plant cell walls, breaking through host passive defense systems, and establishing PHI relationships (Cantarel et al., 2009). The enzymes encoded by the GH, CE, and PL family genes play a role in depolymerizing cell walls (Walton, 1994), and they had only slight differences between the two formae speciales. Considering the errors in gene sequencing and energy prediction, the CAZy species and quantity of the two formae speciales could basically be thought to be consistent at the genome level. Further, 32 and 26 CBM family genes were found in the *S. turcica* f. sp. *sorghi* GD003 and *S. turcica* f. sp. *zeae* Et28A genomes, respectively; the modules of approximately 40 residues in the family genes were unique to the fungi and played a key role in cellulose degradation in plant cell walls (Quentin et al., 2002). Comparison of CAZy annotation results in secreted proteins of the two formae speciales revealed that the content of GHs in *S. turcica* f. sp. *sorghi* GD003 was significantly higher than that in *S. turcica* f. sp. *zeae* Et28A, which might suggest that the former has stronger pathogenic ability than the latter.

Among the secondary metabolites of plant pathogens, melanin and toxin are the two key pathogenic factors in *S. turcica*. The synthesis of these two virulence substances is mainly mediated by PKSs. *PKS1* had been successfully cloned in *B. oryzae* and *C. resinifera* and was found to affect the synthesis of melanin and reduce pathogenicity (Moriwaki et al., 2004; Tanguay et al., 2006). *StPKS* of *S. turcica* f. sp. *zeae* was shown to play a role in the DHN melanin synthesis pathway, and its decreased expression reduced melanin production (Zhang et al., 2011). Moreover, *S. turcica* f. sp. *zeae* Et28A had an additional betaenone biosynthetic gene cluster unlike in *S. turcica* f. sp. *sorghi* GD003; this cluster acted as a phytotoxin that inhibited multiple protein kinases (Patrick and Heimbrook, 1996) and caused significant growth inhibition of *Beta vulgaris* (Haraguchi et al., 1983). This study was the first to identify the core domain of the PKS genes related to melanin and toxin biosynthesis of the two formae speciales of *S. turcica*, and it was found that they had high homology with PKS genes of other pathogenic fungi (common domain of toxin-synthesized PKS genes: KS + AT + DH; common domain of melanin-synthesized PKS genes: KS + AT). Differences in key genes involved in secondary metabolite synthesis have less effect on pathogenic differentiation of the two formae speciales of *S. turcica*.

The determination of the host range in plant pathogens is often closely related to the fungal effectors (Baroncelli et al., 2016). A total of 346 candidate effectors in *S. turcica* were identified by time-course RNAseq, and SIX13-like proteins of *S. turcica* isolated from corn and sorghum were demonstrated to have host-specific polymorphisms (Human et al., 2020). In this study, we first excluded the influence of shared effector protein coding genes of pathogens on host specificity, and only analyzed the expression of specific effector protein-coding genes in *S. turcica* f. sp. *zeae* during the interaction with corn. In the future, further verification of the functions of differential genes is required. During the interaction between plants and pathogens, the activity of hydrolase is conducive to the invasion of the pathogen and the expansion of the disease course. The hydrolytic enzymes related to pathogenicity mainly include cellulase, hemicellulase, pectinase, xylanase, etc. (van den Brink and de Vries, 2011). The significant up-regulation of α-L-arabinofuranosidase encoding gene-A3531 (targeting xylan in plant fibers) during the infection process once again proves that many pathogenic related genes are simultaneously expressed in the interaction, and the time and level of expression determine the pathogenic level of the pathogen to the host (Kim et al., 2016).

Cellulose, an important component of plant cell walls, has a stable structure, which functions effectively to resist pathogen invasion and exogenous stress (Hu et al., 2018). Many cell wall degrading enzymes produced by pathogens cooperate to degrade host cell walls and infect the host (Cooper et al., 1988). Novo et al. (2006) found high levels of activity for endo-1, 4-β-D-glucanase, and β-1, 4-D-glucosidase in invasive *V. dahliae* strains. Furthermore, highly invasive *P. nodorum* strains can produce more cellulase (Lalaoui et al., 2008). The cellulase activity of *S. turcica* f. sp. *zeae* was previously reported to be slightly higher than that of *S. turcica* f. sp. *sorghi*, and it was noted that differences in cell wall degrading enzymes might be one of the reasons for its pathogenic specialization (Tang et al., 2015). The protein encoded by A2464 was annotated as a member of the seventh family of GHs with endo-1, 4-β-D-glucanohydrolase activity (EC 3.2.1.4). This is an important component of cellulase gene and might be a cause of the pathogenicity of *S. turcica* f. sp. *zeae* Et28A. Therefore, we selected the highest expressed *S. turcica* f. sp. *zeae* specific cellulase gene (*StCEL2*) for analysis. In this study, we used gluconolactone to treat *S. turcica* f. sp. *zeae* and analyzed its inhibitory effect on cellulase activity, and our results are consistent with those of Holtzapple et al. (1990). Interestingly, the expression of *StCEL2*, an endo-1, 4-β-D-glucanase coding gene, was upregulated, and increased with increasing concentration of gluconolactone. This is probably due to gluconolactone inhibiting cellulase activity, and the pathogen taking advantage of this stress to invade the host, inducing the expression of related genes (Kou et al., 2014). In summary, gluconolactone can reduce infection rate and pathogenicity by inhibiting the cellulase activity of *S. turcica* f. sp. *zeae*. Furthermore, methods such as gene knockout will be applied to reveal the reasons of two formae speciales infect specific hosts. These results provide useful information for understanding the mechanism of infection and pathogenic differentiation of *S. turcica* f. sp. *zeae*.

## Conclusion

In this study, we reported the genome sequence of *S. turcica* f. sp. *sorghi* and compared it with the number of genes of *S. turcica* f. sp. *zeae* in each functional database. Because of the obvious host specificity of the two formae speciales, we focused on the differences in the coding genes of secreted proteins and secondary metabolites, and pointed out the expression levels of specific effector protein-coding genes in *S. turcica* f. sp. *zeae* at different infection periods. Furthermore, the close relationship between cellulase and pathogenicity of *S. turcica* f. sp. *zeae* was determined by the inhibitory effect of enzyme activity, and it was clear that cellulase was one of the important factors of its pathogenicity. In summary, our results provide a novel ideas for studying the interaction between pathogens and the host, and lay a strong foundation for further knockout of cellulase genes and mining of pathogenicity-related genes. Obviously, our data improve the understanding of important pathogens of *S. turcica*, increase the genomic information of *S. turcica* f. sp. *sorghi*, and contribute to the study of pathogenic mechanisms.

## Data availability statement

The raw sequence reads of *Setosphaeria turcica* f. sp. *sorghi* have been deposited in GeneBank under the accession number PRJNA860778.

## Author contributions

ZM, BL, and ZG conceived and designed the experiments. ZM, YH, ZZ, and XL performed the experiments and analyzed the data. ZM drafted the manuscript, which was critically revised by YX. All authors contributed to the article and approved the submitted version.

## Funding

This project was supported by the National Key Technology Research and Development Program of China (2018YFD0300307, 2017YFD0300704, and 2016YFD0300704).

## Conflict of interest

The authors declare that the research was conducted in the absence of any commercial or financial relationships that could be construed as a potential conflict of interest.

## Publisher’s note

All claims expressed in this article are solely those of the authors and do not necessarily represent those of their affiliated organizations, or those of the publisher, the editors and the reviewers. Any product that may be evaluated in this article, or claim that may be made by its manufacturer, is not guaranteed or endorsed by the publisher.
