# Corrigendum: Comparative genomic analysis reveals cellulase plays an important role in the pathogenicity of *Setosphaeria turcica* f. sp. *zeae*

**DOI:** 10.3389/fmicb.2022.1064424

**Published:** 2022-11-16

**Authors:** Zhoujie Ma, Yufei Huang, Zhaoran Zhang, Xiaodi Liu, Yuanhu Xuan, Bo Liu, Zenggui Gao

**Affiliations:** ^1^Institute of Plant Immunology, College of Plant Protection, Shenyang Agricultural University, Shenyang, China; ^2^College of Life Sciences, Yan'an University, Yan'an, China

**Keywords:** *Setosphaeria turcica*, formae speciales, pathogenicity, genome sequencing, gene function, comparative genome, cellulase activity

In the published article, there was an error in Supplementary Figures 1–6 and Supplementary Tables 1–7. The correct Supplementary material only contains 2 Supplementary Figures, 3 Supplementary Tables, and 3 Supplementary Fasta files.

The authors apologize for this error and state that this does not change the scientific conclusions of the article in any way. The original article has been updated.

## Publisher's note

All claims expressed in this article are solely those of the authors and do not necessarily represent those of their affiliated organizations, or those of the publisher, the editors and the reviewers. Any product that may be evaluated in this article, or claim that may be made by its manufacturer, is not guaranteed or endorsed by the publisher.

