# Supplementary material for: Comparative genomic analysis reveals cellulase plays an important role in the pathogenicity of Setosphaeria turcica f. sp. zeae
Source: Front Microbiol. 2022 Jul 22;13:925355. doi: 10.3389/fmicb.2022.925355 (PMC9355644; doi:10.3389/fmicb.2022.925355)
Supplement: Supplementary file 1 [file Data_Sheet_1.zip › Supplementary Figure S1.doc]

**SUPPLEMENTARY FIGURE 1︱**Correlation analysis of GC content and sequencing depth in *Setosphaeria turcica* f. sp. *sorghi* GD003. Note: the abscissa indicates the GC content, the ordinate indicates the sequencing depth, the right is the sequencing depth distribution, and the upper is the GC content distribution.
