# Supplementary material for: Comparative genomic analysis reveals cellulase plays an important role in the pathogenicity of Setosphaeria turcica f. sp. zeae
Source: Front Microbiol. 2022 Jul 22;13:925355. doi: 10.3389/fmicb.2022.925355 (PMC9355644; doi:10.3389/fmicb.2022.925355)
Supplement: Supplementary file 1 [file Data_Sheet_1.zip › Supplementary Figure S2.doc]

**SUPPLEMENTARY FIGURE 2︱**Functional description and classification of secreted proteins encoded by the whole genomes of the two formae speciales. GD003 specific: *Setosphaeria turcica* f. sp. *sorghi* GD003-specific genes relative to those in *S. turcica* f. sp. *zeae* Et28A, Et28A specific: *S. turcica* f. sp. *zeae* Et28A-specific genes relative to those in *S. turcica* f. sp. *sorghi* GD003, Common: genes shared by *S. turcica* f. sp. *sorghi* GD003 and *S. turcica* f. sp. *zeae* Et28A.
