# Supplementary material for: Comparative genomic analysis reveals cellulase plays an important role in the pathogenicity of Setosphaeria turcica f. sp. zeae
Source: Front Microbiol. 2022 Jul 22;13:925355. doi: 10.3389/fmicb.2022.925355 (PMC9355644; doi:10.3389/fmicb.2022.925355)
Supplement: Supplementary file 1 [file Data_Sheet_1.zip › Supplementary Table S1.doc]

**SUPPLEMENTARY TABLE 1︱Analysis of the PHI phenotypic mutation types of the two formae speciales.**

| **PHI Phenotype classification** | ***S. turcica* f. sp. *sorghi* GD003** | ***S. turcica* f. sp. *zeae* Et28A** |
| --- | --- | --- |
| Chemistry target: resistance to chemical | 3 | 3 |
| Effector (plant avirulence determinant) | 2 | 2 |
| Increased virulence (hypervirulence) | 28 | 22 |
| Lethal | 38 | 35 |
| Loss of pathogenicity | 88 | 86 |
| Reduced virulence | 298 | 259 |
| Unaffected pathogenicity | 339 | 266 |
