# Supplementary material for: Comparative genomic analysis reveals cellulase plays an important role in the pathogenicity of Setosphaeria turcica f. sp. zeae
Source: Front Microbiol. 2022 Jul 22;13:925355. doi: 10.3389/fmicb.2022.925355 (PMC9355644; doi:10.3389/fmicb.2022.925355)
Supplement: Supplementary file 1 [file Data_Sheet_1.zip › Supplementary Table S2.doc]

**SUPPLEMENTARY TABLE 2︱Statistics of CAZys in secreted proteins of the two specialized strains.**

| **Family** | ***S. turcica* f. sp. *sorghi* GD003** | ***S. turcica* f. sp. *zeae* Et28A** | **Putative function** |
| --- | --- | --- | --- |
| **PL** | 12 | 11 |  |
| PL1 | 4 | 4 | pectate lyase, exo-pectate lyase |
| PL3 | 5 | 4 | pectin lyase |
| PL4 | 3 | 3 | rhamnogalacturonan lyase |
| **GH** | 104 | 86 |  |
| GH3 | 6 | 4 | beta-glucosidase, etc. |
| GH5 | 7 | 4 | endo-beta-1,4-xylanase, etc. |
| GH11 | 5 | 4 | endo-beta-1,4-xylanase, etc. |
| GH16 | 5 | 5 | beta-agarase, etc. |
| GH28 | 6 | 2 | polygalacturonase, etc. |
| GH43 | 5 | 5 | beta-xylosidase, etc. |
| others | 70 | 62 |  |
| **CE** | 21 | 18 |  |
| CE1 | 3 | 3 | acetyl xylan esterase, etc. |
| CE5 | 10 | 8 | acetyl xylan esterase, cutinase |
| CE8 | 2 | 2 | pectin methylesterase |
| CE16 | 3 | 2 | acetylesterase, active on various carbohydrate acetyl esters |
| others | 3 | 3 |  |
| **AA** | 44 | 34 |  |
| AA3 | 11 | 9 | cellobiose dehydrogenase, glucose 1-oxidase |
| AA9 | 19 | 18 | copper-dependent LPMOs |
| others | 14 | 7 |  |
| **GT** | 3 | 3 |  |
| GT24 | 1 | 1 | UDP-Glc: glycoprotein alpha-glucosyltransferase |
| GT25 | 1 | 1 | beta-1,3-glucosyltransferase, etc. |
| GT32 | 1 | 1 | alpha-1,6-mannosyltransferase, etc. |
| **CBM** | 32 | 26 |  |
| CBM1 | 11 | 12 | cellulose-binding function |
| CBM13 | 4 | 2 | modules of glycoside hydrolases and glycosyltransferases |
| CBM18 | 5 | 3 | chitin-binding function |
| CBM35 | 4 | 3 | conservative modules in xylanolytic enzyme |
| others | 8 | 6 |  |
