# Supplementary material for: Comparative genomic analysis reveals cellulase plays an important role in the pathogenicity of Setosphaeria turcica f. sp. zeae
Source: Front Microbiol. 2022 Jul 22;13:925355. doi: 10.3389/fmicb.2022.925355 (PMC9355644; doi:10.3389/fmicb.2022.925355)
Supplement: Supplementary file 1 [file Data_Sheet_1.zip › Supplementary Table S3.doc]

**SUPPLEMENTARY TABLE 3︱AntiSMASH prediction of secondary metabolic-related genes shared by the two formae speciales.**

| **Gene cluster** | ***S. turcica* f. sp. *sorghi* GD003** | ***S. turcica* f. sp. *zeae* Et28A** |
| --- | --- | --- |
| PKS | 20 | 15 |
| NRPS | 7 | 6 |
| PKS-NRPS | 4 | 2 |
| Indole | 2 | 1 |
| Terpene | 5 | 4 |
| Lantipeptide | 1 | 0 |
| Other | 7 | 5 |
| Total | 46 | 33 |
